# Effects of Speaker Emotional Facial Expression and Listener Age on Incremental Sentence Processing

**DOI:** 10.1371/journal.pone.0072559

**Published:** 2013-09-06

**Authors:** Maria Nella Carminati, Pia Knoeferle

**Affiliations:** 1 Department of Linguistics, University of Bielefeld, Bielefeld, Germany; 2 Language and Cognition Group, Cognitive Interaction Technology Excellence Cluster, University of Bielefeld, Bielefeld, Germany; University of Melbourne, Australia

## Abstract

We report two visual-world eye-tracking experiments that investigated how and with which time course emotional information from a speaker's face affects younger (*N* = 32, Mean age  = 23) and older (*N* = 32, Mean age  = 64) listeners’ visual attention and language comprehension as they processed emotional sentences in a visual context. The age manipulation tested predictions by socio-emotional selectivity theory of a positivity effect in older adults. After viewing the emotional face of a speaker (happy or sad) on a computer display, participants were presented simultaneously with two pictures depicting opposite-valence events (positive and negative; IAPS database) while they listened to a sentence referring to one of the events. Participants' eye fixations on the pictures while processing the sentence were increased when the speaker's face was (vs. wasn't) emotionally congruent with the sentence. The enhancement occurred from the early stages of referential disambiguation and was modulated by age. For the older adults it was more pronounced with positive faces, and for the younger ones with negative faces. These findings demonstrate for the first time that emotional facial expressions, similarly to previously-studied speaker cues such as eye gaze and gestures, are rapidly integrated into sentence processing. They also provide new evidence for positivity effects in older adults during situated sentence processing.

## Introduction

The study of context effects on language processing has been a major research topic in psycholinguistics, and over the years findings in this area have influenced theories of language processing in an important way. In the early days, the focus of investigation was on effects of the immediately preceding *linguistic* context; however, since the development of the visual-world paradigm in the mid-nineties, psycholinguists have had at their disposal a powerful tool to investigate another potentially very rich source of context effects on language processing, i.e., the concurrent visual world (for a review see [1]–[2]).

In the visual-world paradigm, participants listen to spoken sentences about objects and events in a scene, and we record their eye movements to these objects and events (e.g., [3]–[5]). Typically in this paradigm, participants look at those parts of the scene that are mentioned in the sentence; this reflects the process of understanding the sentence in relation to the concurrent visual context [1], [6]. Because the record of fixations on the scene is synchronised with the onset of the words in the unfolding utterance, the visual world paradigm provides precious information about the time course with which different types of information from the visual context affect a listener's eye movements during incremental sentence comprehension. Indeed, findings from the visual world paradigm have shown that a supporting visual context exerts a rapid effect on the resolution of syntactically ambiguous sentences, eliminating in practice the garden path that is observed when these sentences are read in isolation (e.g., [5], [7]). Moreover information such as an object's size, color [8], or shape [9], depicted clipart events [7], real-world action events [10], action affordances [11], and the spatial location of objects [12] are all rapidly integrated during sentence comprehension and can affect a listener's visual attention within a few hundred milliseconds.

In more recent years research into visual context effects on language processing has widened considerably in scope to cover subtle aspects typical of more naturalistic, visually-situated dialogue interactions. One area of investigation has been how visually-perceivable speaker-based cues such as eye gaze affect language processing. Results suggest that a speaker's gaze is integrated incrementally into language processing by listeners (e.g., [13]–[15]). For example, during a collaborative task, listeners shifted their gaze to an object in their workspace soon after they had seen the speaker attend to the corresponding object in her workspace [14]. Overall, when a speaker inspects and mentions objects, listeners rapidly align their gaze with the gaze of the speaker [16]–[17]. Richardson and Dale [16] found that closer coordination of a listener's eye movements with the speaker's gaze resulted in better listener performance on a comprehension test. Kreysa and Knoeferle [18] further showed that listeners were able to anticipate thematic roles earlier when speaker gaze was available, compared to when it was not.

Gaze is not the only kind of information provided by a speaker's face during verbal interaction. A further useful speaker-based cue is gestures; indeed, the gestures of a speaker can influence both syntactic disambiguation and semantic interpretation in the listener within a few hundred milliseconds: Iconic gestures can rapidly influence semantic interpretation, as reflected by event-related brain potentials [19], and beat gestures, syntactic structuring [20]. Thus, information from objects, actions, a speaker's gaze, and gestures all have been shown to rapidly affect a listener's visual attention and online sentence comprehension.

Another potentially powerful cue in sentence comprehension is the speaker's emotional facial expression. However, to the best of our knowledge there is to date no study that has examined effects of a speaker's facial expression on spoken sentence comprehension. Moreover, few studies have examined how speaker-based information can inform language comprehension when language is ‘visually situated’ (i.e., when it is about objects and actions in the visual context). Additionally, evidence for visual context effects in language comprehension comes almost exclusively from studies with young adults (ca. 19–31 years). By contrast, the extent to which visual context affects sentence comprehension in young children (e.g., [21]; but see, e.g., [22]–[24]), and in older adults is less clear. The present paper addresses these open issues and examines (a) the time course with which a speaker's emotional facial expression can influence a comprehender's visual attention to target pictures during spoken sentence comprehension; and (b) the nature of this influence in young versus older adults. We base our hypotheses about how a speaker's emotional expression might influence sentence interpretation on how emotional faces, pictures, and words are processed also in young compared with older adults. Below we review relevant findings and summarize the hypotheses for the present study (facial priming of sentence comprehension).

### Emotional faces and other emotional stimuli enjoy privileged attention

In his pioneering work, Ekman [25] proposed a set of six basic universal emotions associated with distinct facial expressions (happiness, sadness, fear, anger, disgust, and surprise). This classification and associations of basic emotional meaning with facial expressions have been widely tested in emotion research [26]–[29]. Although emotions can be expressed by means other than the face (for example, through body posture, gestures or voice modulation), the face has a privileged status as a vehicle of emotion expression ([30], but see [31]). One common finding is that emotional faces, such as happy or angry ones, are attended to faster and are processed more deeply than neutral ones [32]–[34]. Based on Ekman's proposal and these related findings, face-meaning associations could, in principle, be used by a speaker to strengthen the meaning of her utterances. It is thus not implausible to assume that a spoken statement of anger uttered with a correspondingly angry facial expression will have a bigger impact on the listener than the same statement uttered with a neutral facial expression.

The same attention advantage enjoyed by emotional faces (compared to non-emotional, neutral ones) exists for emotional stimuli such as pictures, sounds, and language [35]–[36]. For example, in the domain of emotional word processing, analyses of event-related brain potentials revealed an enhancement of early cortical responses to emotional (positive and negative) compared with neutral words [37]. Furthermore, in a post-experiment recall task, emotional words were recalled better than neutral ones (see [38], for a similar result). The memory advantage of emotional over neutral words extends to other stimuli such as pictures (e.g., [39]–[40]). Overall thus, emotional stimuli (faces, words, and pictures) seem to receive more attention than neutral stimuli.

### Emotion processing in young vs. older adults

Interestingly, the processing of emotion, and specifically of positive and negative valence, changes across the life span (for a review, see [41]–[42]). One manifestation of this is that with advancing age people increasingly prioritise positive over negative information (‘positivity effect’). According to the proponents of the socioemotional selectivity theory (for a review, see [43]–[44]), this change arguably relates to older people's realization that their time is limited and an associated desire to optimize emotionally-satisfying experiences. For example, when presented with pairs of pictures consisting of a neutral face and a positive (happy) or negative (sad or angry) face, older people spent less time looking at the negative face than the positive one; in other words, they displayed an attentional bias away from the negative and towards the happy facial expressions [45]–[46]. Younger people, on the other hand, show no preference [46], or prefer negative faces [47]. Positivity effects have also been found in memory tasks, with older adults recalling a greater number of positive than negative pictures [48], facial expressions [46], or long-term life events [49]. There is also recent brain-imaging evidence for the positivity effect [50]–[51]. In recent years, there has been some discussion among researchers about the proper characterization of the positivity effect and the experimental conditions under which it can be observed. This discussion has led to a broadening of the definition, which now includes not only an increased focus on positive information, but also a reduced focus on negative information by older adults (see especially [44],[50],[52]; also [48] Expt 2, [53]–[55]). Because the positivity effect concerns the relative difference in attention to positive as opposed to negative information between older and younger people, it is consistent not only with a pattern of results in which older people show a positivity bias and young ones a negativity bias, but also with a pattern in which younger adults show a negativity bias and older adults show no bias, and a pattern in which younger adults show no bias and older ones show a positivity bias [50], [52].

### Emotion priming

Emotional stimuli and, in particular, faces can also influence how other stimuli (e.g., words, pictures) are processed. When faces occur together with other contextual cues, the latter can heavily influence the recognition of facial emotional (for a recent review, see [56]). For example, emotional faces are recognized better in the presence of a congruent affective body posture or scene context [57]–[58], or by congruent sentential prosody [59].

Emotional stimuli can also influence the processing of subsequent information. This influence has been demonstrated robustly in many studies on so-called emotion priming, a type of priming using stimuli with inherent emotional valence [60]–[62]. Typically, responses to a target stimulus are facilitated (i.e., faster) when prime and target have the same emotional valence (e.g., positive-positive, negative-negative) as opposed to when they have opposite valence (e.g. positive-negative). In experimental settings, emotion priming emerges both within modality and category (e.g., two faces, pictures or words) and across modalities. For example, priming can occur when the prime is a picture and the target is a facial expression ([63] Expt 2); when the prime is a picture or facial expression and the target is a word (see among others, ([63] Expt 1 and 4, [64]–[66]) or when the prime is a word and the target a facial expression ([63] Expt 2). In sum, emotional stimuli can influence and facilitate the processing of similar-valence stimuli that are presented simultaneously or subsequently.

### Current study: Emotion priming of sentences in young vs. older adults

To the extent that the just-mentioned findings on emotion priming from faces to words extend to visual attention and *sentence* processing, a smile on a speaker's face should facilitate the processing of a positive sentence (compared to a negative sentence) on the part of the listener. To our knowledge, no research so far has investigated emotion priming of whole sentences (as opposed to isolated words) using emotional facial expressions. In the current study, we used the visual-world paradigm to examine the time course of emotion priming in sentence processing. Participants listened to sentences which related to visual material displayed on a computer screen. Before hearing a sentence, they saw either a smiling or a sad face, which they were told was the face of the speaker of the sentence they were going to hear next (thus simulating a speaker-hearer scenario). Then two emotional pictures from the International Affective Picture System database (IAPS, [67]), one positive and one negative, were displayed side by side on the screen. After that a sentence about one or the other picture was played out while the pictures remained on the screen; accordingly, the sentence had also a positive or negative emotional content. The speaker's facial expression could have the same emotional valence as the sentence, or the opposite valence. Participants' eye movements to the display on the monitor while they listened to the sentence were recorded. In line with the usual findings from the visual world paradigm, when participants begin processing the sentence, we expect them to start directing their looks to the IAPS picture the sentence is about and to increasingly inspect this picture as the words of the sentence unfold over time.

#### Face-sentence and face-picture priming effects: time course

The more interesting question concerns how the facial prime affects the processing of the sentence as revealed by the fixations that people make on the pictures during sentence processing. Recall that responses to a target stimulus are facilitated (i.e., faster) when a prime stimulus is emotionally congruent with the target than when it is not (see above). In our visual world experiment the primary response is a series of eye fixations that participants make on the IAPS pictures as the sentence unfolds. If the facial prime affects (i.e., primes) the processing of the sentence, listeners should look more and longer at the target picture (the picture that is described by the sentence) when the emotional face is valence-congruent with the sentence than when it is not. More and longer target picture fixations have been found to reflect priming in other language comprehension priming experiments [68]–[69]. Therefore we refer to this pattern of eye behavior as face-sentence priming. According to standard assumptions about the relationship between eye fixations and language processing in the visual context [1]–[2], this pattern shows that pre-activated emotional information (faces) can guide visual attention to congruous emotional information (pictures). One interpretation of this pattern might be that it reflects more in-depth processing of the attended information. It has been interpreted as the counterpart of the facilitatory effects observed in priming experiments measuring button-press RTs. Thus from now on we will assume that face-sentence priming behavior reflects facilitation and use the terms ‘facilitation’ and ‘face-sentence priming’ interchangeably to refer to such behavior.

As regards the time course of this facilitation, as already mentioned, earlier findings suggest that emotional information enjoys privileged attention, so facilitation effects should occur from the early stages of processing the sentence. Alternatively, considering the specifics of our experimental task, facilitatory effects may not surface until later or not occur at all during the processing of the sentence. This is because for facilitation to take place perceivers need to integrate cues from the visual, linguistic and emotional modalities and this may be a complex task to perform on the fly.

Another pattern of priming could also be observed, that is, a preference to look at the picture which is emotionally congruent with the face (face-picture priming). This would manifest itself in more and longer looks to the face-congruent picture. It could occur before face-sentence facilitation, as it does not require the sentence but only information from the face and the pictures. Indeed, this would resemble effects in emotion priming research that did not manipulate language (e.g., [63]).

In our experiment, participants also had to verify post-sentence with a button press whether the face matched or did not match the sentence. So our paradigm permits us to trace the time course of facial emotion priming during sentence comprehension up to later post-comprehension responses (see also [70]–[71], for a related approach concerning action and speaker gaze effects). If facial emotion priming occurs once, during sentence comprehension, and then fades away, we should see no effects in post-sentence response latencies and accuracy in the verification task. Alternatively, priming through the speaker's facial emotional expression has a longer-lasting effect, as one might expect based on prior findings with other types of information in visual context (e.g., [10]). If so, post-sentence responses should correlate with eye tracking results. Specifically, in line with our discussion in the previous paragraphs of this section, facilitatory effects in eye-tracking behavior in face-congruent conditions (i.e., more and longer looks) should correspond to facilitatory effects in response and accuracy scores in these conditions (i.e. shorter RTs and higher accuracy).

#### Young versus older adults

On the basis of the observed age-related differences in emotion processing, younger and older adults may differ in how they integrate the information from a negative or positive face with the processing of a negative or positive target sentence and the corresponding picture, that is, it is plausible to expect priming from the face to be modulated by age. According to socio-emotional selectivity theory, we should observe a positivity effect. In particular, because older adults focus more on positive than negative information, for face-sentence priming we would expect a positive face to be a more effective prime for a positive sentence than a negative face is for a negative sentence, and the opposite for younger adults. This would result in facilitation occurring only for positive sentences and not for negative sentences (or greater facilitation with positive than negative sentences) in older adults, and the opposite for younger adults. But other patterns would also be compatible with the broader definition of the positivity effect (see above, [44], [50], [52]); for example, a pattern where younger people show equal facilitation for positive and negative sentences (or no facilitation with either), and older ones only for positive sentences, or a pattern where older people show equal facilitation for positive and negative sentences (or no facilitation with either), and younger ones only for negative ones. A result where older people display reduced facilitation for negative sentences compared to younger people would also be compatible with a positivity effect [50]. These modulations by age can be predicted for face-sentence priming and face-picture priming in the eye-movement behavior, and for the verification response times and accuracy. They should be manifest in the presence of a significant interaction of age with face-sentence and face-picture priming [50], [44]. To obtain a more detailed picture of age-related differences, we will also compare young and older people's picture bias (i.e., a preference to look at the positive or negative picture) irrespective of face and sentence manipulation. In fact, according to socio-emotional selectivity theory, one could predict that older people would, to varying degrees, tend to look more at the positive than negative picture independent of the face and sentence manipulations. A positivity effect in this measure should result in age differences in picture inspection. As for possible age differences in the time course of the expected effects, older people may be as fast as the younger ones in responding to the face and sentence manipulation; alternatively, because of their age and concurrent cognitive decline, they may show a delay [72]–[73].

## Methods

### Participants

The participants were 32 older (60–72 years, *M* = 64.37, *SD* = 3.57) and 32 younger (19–29 years, *M* = 22.90, *SD* = 2.73) adults. Older adults (15 female, 18 male) were recruited through advertisements posted in the university and in other public places of the city of Bielefeld, Germany. The younger participants (20 female, 12 male) were students at the University of Bielefeld. Younger and older participants received a monetary reward for their participation in the experiment.

### Ethics statement

As the University of Bielefeld did not yet have an official institutional Ethical Review Board, at the time of grant application, the issue of ethics approval was raised with our sponsor (The German Research Foundation, DFG), who replied that under their standard procedure for psycholinguistic research, obtaining ethics approval would not be necessary for our research (a copy of the communication is available from PLOS). From our part, all the necessary steps were taken to conduct the research following the guidelines laid down in the Declaration of Helsinki. Before the experiment all participants had to read an Information Sheet in which they were informed about the experiment and the tasks involved, about the potential risks and discomforts (none known, since we perform non-invasive behavior experiments), and about data treatment. After reading the information sheet and receiving any further clarifications from the experimenter, participants signed a written Informed Consent form, in which it was stated that they could discontinue the study at any time if they wished to do so. During the experiment utmost care was taken to ensure that participants were feeling comfortable and well. After the experiment participants were debriefed and received an answer to any questions they had. Copies of the Information Sheet and the Informed Consent form are available from PLOS.

The categorization of participants by age was exclusively for the purpose of the experimental manipulation of our study. We controlled for key cognitive variables with the administration of cognitive tests (see notes to Table 1).

**Table 1:** Cognitive test results and demographic characteristics of the younger and older adults.

Characteristic	Younger	Older
Age range	18–30	60–80
Mean age in years	22.9 (2.7)	64.4 (4.5)
Animal naming^a^	27.44 (5.76)	28.71 (5.86)
Picture completion^b^	4.37 (0.83)	3.68 (1.15)*
Digit Symbol^b^	82.37 (11.78)	67.62 (10.17)*
Word naming^c^	12.94 (4.58)	12.91 (3.80)
Digit span^b^	17.41 (3.40)	16.68 (3.46)
Similarities^b^	13.56 (1.74)	13.60 (2.09)
BMIS scores^d^	8.34 (6.46)	10.50 (8.72)
Male/female (*n*)	12/20	18/15

*Note*. Standard deviations are in parentheses.

a Task: Name as many animals as possible, time allowed: 1 min.

b latest German version of the Wechsler Adult Intelligence Scale (WAIS), [79]–[80].

c Task: Name as many words as possible starting with the letter ‘l’, time allowed: 1 min.

d Brief Mood Introspection Scale questionnaire (BMIS, [81]) translated into German. The mood rating for each participant was obtained by subtracting the scores for negative mood from those of positive mood.

* Younger and older adults' means differ significantly (*t* test, *p*<.05).

### Materials

#### Pictures of prime faces

Fifteen students from the University of Bielefeld were recruited for photographs of facial expressions. They were asked to simulate a neutral (neutral faces were used in filler items), a sad and a happy expression. Most found the production of a sad face difficult, and were encouraged to silently re-experience unpleasant events so as to evoke the corresponding negative facial expression. The portraits were taken frontally against a white background at a distance of about 1 metre from the camera and with the use of the flash. The photographic sessions yielded 15 face triplets (one negative expression, one positive expressions and a neutral one). Eighteen students that did not take part in the eye-tracking experiment (Mean age  = 24.7, *SD* = 2.74) rated these faces on a nine-point scale (1 =  very negative, 9 =  very positive). Of the initial 15 photo sets we selected the four best sets (2 male and 2 female). Item selection was based on maximal difference in ratings between the valenced (positive and negative) and the neutral expressions. While the recognition of happy faces is often straightforward, sad faces are arguably more ambiguous, and thus more difficult to recognize [26]. Maximizing the valenced-to-neutral differences is thus important to ensure rapid recognition of facial valence. The best two (1 male and 1 female) of these four sets were assigned to the experimental items and the remaining two to the filler items. The face stimuli of the experimental items, with their mean rating and *SD*'s, are available in Supporting Information S1. These two individuals have given written informed consent, as outlined in the PLOS consent form, to publication of their photographs.

#### IAPS pictures

We selected a total of 84 pairs of images from the IAPS database [67]. These were allocated to the experimental items (28 pairs of opposite-valence pictures; negative-positive with at least one human depicted) and to the filler items (56 pairs). Selection of the negative and positive image for experimental item pairs was based on the valence ratings from the original norming sample of both men and women (see [67], Table 1; 1 =  very negative to 9 =  very positive). For the negative images, we chose IAPS photographs with ratings towards the negative end of the scale but without extreme negative ratings as these might be too unpleasant to show to participants (negative ratings: 2.42 to 5.07; *M* = 3.46, *SD* = 1.69). Positive images ranged from 5.51 to 8.22 (*M* = 7.19, *SD* = 1.55; positive-negative mean difference: 3.73). As arousal has been shown to interact with valence in emotion priming [75]–[76], negative and positive images were controlled for arousal score [74] (negative picture mean arousal score: 4.73, *SD* = 2.06; positive picture mean arousal score 4.94, *SD* = 2.27). A paired *t*-test on the arousal scores of negative and positive pictures was not significant (*t* (27)  = −.84, *p* = .41). We provide a list of the experimental images and their IAPS number in Supporting Information S2.

The filler items consisted of 56 IAPS image pairs from the mid-valence range (ratings: 3.5–6.5). These images featured a range of themes such as people, household objects, natural scenes and objects. In pairing photographs, we maximized their visual similarity on the following criteria for all items: the number of people in the image (one person, a pair, a small group, or a large group), the location of the people within the image, the orientation of the people (facing others vs. facing away, similar body posture, etc.), the brightness of the image, and the predominant colours. This was done to minimize attentional bias for one (vs. the other) picture due to visual characteristics.

#### Construction of sentences

**Figure 1:**
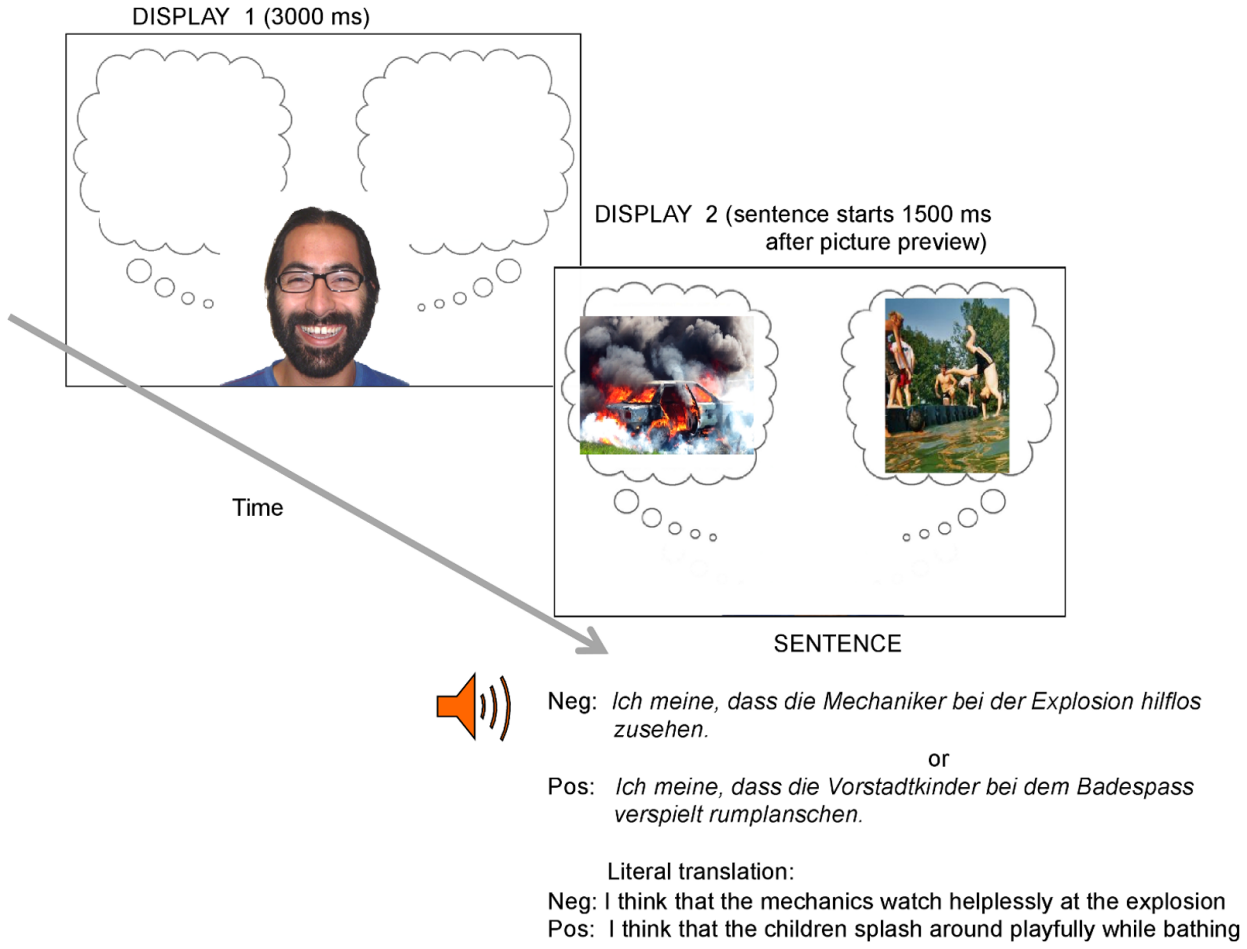
Sequence of events in an experimental trial. For copyright restrictions the IAPS pictures have been replaced in this figure with similar pictures downloaded from the web. (Sources: left picture: http://www.sxc.hu/photo/1240763; right picture: http://www.sxc.hu/photo/48144, with thanks to Stefan Wagner, trumpkin.de).

We constructed 56 experimental German sentences (one for each of the two images of the 28 experimental item-picture pairs). All started with a main clause containing a verb of opinion in the first person singular (e.g., *I think/believe/am of the opinion that*…). This was followed by a subordinate clause referring to the event depicted in the picture. In 24 items, the subordinate clause began with a subject noun phrase (NP1), followed by an object noun phrase (NP2), an adverb (Adv) and a final finite verb (Verb). The remaining 4 items had the same structure except that the NP2 object was part of a prepositional phrase. Within each experimental item pair, the sentences for the positive and negative image contained the same number of syllables for 25 items, while the remaining three differed in length by one syllable. Examples of the positive and negative sentence for an item are given in Figure 1. Sentences were matched in lemma frequency of nouns and adverbs (CELEX, [77]). An additional feature of the semantic content of the experimental sentences was their emotional gradedness. The initial main clause was emotionally neutral and identical between positive and negative sentences of an item. The initial noun of the embedded sentence (NP1) was relatively neutral. However, the following noun (NP2) and the adverb had high emotional connotations, in line with the picture associated with the sentence (i.e., strongly positive in the positive sentences and strongly negative in the negative sentences). The experimental sentences associated with each of the IAPS pictures are given in Supporting Information S2.

For the 56 filler item picture-pairs, we constructed sentences that matched only one of the pictures. About one third of the sentences had the same structure as the sentences for the experimental items (e.g., *I think that*…), while the rest consisted of main clauses. The content of half of the filler sentences (28) was neutral (e.g., *I think that the judge has worked abroad previously*), 14 contained at least one positive word (e.g., *The talented artist is drawing the nice portrait*) and the remaining half one negative word (e.g., *It is obvious that the weather will be unbearable today*).

#### Recording of the sentences

The sentences were recorded by four native speakers of German, two female and two male. The speakers were given example sentences and asked to utter them in as neutral an intonation as possible and at a relatively low pace, leaving a pause between phrases. Each speaker provided two recordings and the second recording was edited to produce individual sound files of the sentences to be used in the experiment. Two speakers, one female and one male, were assigned to the experimental sentences. Then the sound files of the two sentences associated with an experimental picture pair were edited using professional sound editing software, to ensure that the onsets of the critical words (NP1, NP2, Adverb) occurred exactly at the same point in time from sentence start in the positive and corresponding negative sentence (to achieve this, pauses were shortened or breaks between words were lengthened slightly as necessary). The synchronization of the onsets of the critical words within each experimental pair of sentence was done to eliminate possible confounds caused by the temporal misalignment of the critical words during sentence processing. The onsets of the critical words for each item and condition were recorded for later synchronisation of eye movements. Table 2 gives the cue point means (in ms) of the onsets of NP1, NP2, Adverb and Verb from the beginning of the sentence.

**Table 2:** Cue point means (in ms), measured from the start of the sentence. Standard Deviations (*SD*) in parentheses.

Cue points	Positive sentence	Negative sentence
NP1 onset	1848 (341)	1848 (339)
NP2 onset	3148 (332)	3148 (339)
Adverb onset	4466 (488)	4466 (495)
Verb onset	5189 (512)	5191 (523)

#### Experimental design

Each of the 28 experimental picture pairs was combined with (a) a prime face photograph of negative or positive valence and with (b) a sentence describing the positive or the negative picture, to produce the 4 experimental conditions given in Table 3.

**Table 3:** Experimental conditions of the experiment.

	Prime face	Sentence	Prime & sentence match in valence
(a)	Negative	positive	No
(b)	Negative	negative	Yes
(c)	Positive	positive	No
(d)	Positive	negative	Yes

Four lists were constructed according to a Latin square design. This ensured that an experimental item only appeared in one condition in each list. In half of the experimental items (*N* = 14) in a list, the prime face and the sentence matched in valence, while in the remaining half they mismatched (Table 3).

The 56 filler items, consisting of a picture pair, and a sentence describing only one of the pictures, were combined with 28 neutral prime faces, 14 positive prime faces and 14 negative prime faces and were added to each of the experimental lists. As mentioned earlier, the content of the filler sentences was controlled: 28 were neutral, 14 positive and 14 were negative (however all the filler pictures were neutral). This meant that, for each filler item (i.e., the prime face plus target picture/sentence combination) it was possible to say whether the prime face and the sentence matched in valence or not. As we explain later, participants had to provide this judgment for all (filler and experimental) items in the experiment. Filler prime faces (neutral, negative, positive) were assigned to filler sentences/pictures so that participants during the experiment (considering experimental and filler trials as a whole) encountered an equal number of match and mismatch cases. Before the experiment, each list was pseudo-randomized with the constraint that experimental items were separated from each other by at least 2 fillers. Every participant saw a different randomized list.

#### Assignment of speakers/faces to experimental items

The 4 speakers were assigned to the 4 prime faces (2 female, 2 male), with 1 male being used for half of the experimental items (14) and 1 female for the other half (14). The Latin square design applied to the experimental items resulted in half of the items (14) appearing with positive prime faces and the other half (14) with negative ones, distributed equally between the two experimental speakers/faces. Each of the two experimental speakers was also used in 7 filler items, with an associated neutral face. The two remaining speakers (one female, one male) were each assigned to the rest of the filler items (21 each), and each speaker was associated with 7 neutral, 7 positive and 7 negative facial expressions. Overall, this design ensured that in the course of the experiment, participants saw an equal number of prime faces in the neutral, positive and negative condition (i.e., 28 of each), and that each speaker/face was seen with a neutral, sad and happy facial expression for an equal number of times (7 times for each expression).

#### Procedure

The experimental session started with the collection of demographic details from the participants, informed consent, and with the administration of some cognitive tests and of a mood questionnaire (please see the notes to Table 1 for details of these tests).

Eye movements were recorded using an SR Research Eyelink 1000 Desktop head-stabilised eye tracker (SR Research, Mississauga, Ontario, Canada). After the administration of the cognitive tests, the eye tracking session started with a series of eight practice trials to familiarize participants with the task. Participants were told that the study investigated language comprehension in relation to a visual display on the computer screen. In particular, we told them that they would first see the face of a person who was thinking about something and was about to speak, and after that they would hear him/her utter a sentence which described a picture that was on the screen. The task was to look, listen and understand the sentence, and decide whether the valence of the face matched the valence of the sentence (“Does the face match the sentence?”) by pressing one of two buttons. Participants had to answer verification questions on all trials. The sequence of events in an experimental trial is illustrated in Figure 1. The person whose face is depicted in Fig. 1 has given written informed consent, as outlined in the PLOS consent form, to publication of this photograph.

A trial started with a display (Display 1) showing a smiling or a sad face (the prime face) with empty thought bubbles on either side (Fig. 1). Display 1 remained on the screen for 3000 ms. After Display 1 disappeared, two pictures depicting a happy or a sad event respectively appeared on the screen side by side inside the thought bubbles (Display 2). After 1500 ms from the onset of Display 2 a recording of the sentence was played out over the loudspeakers. The sentence was about one of the pictures. Participants were told to listen to the sentence and to answer by pressing one of two buttons as to whether the face matched the sentence. Display 2 remained on the screen for 1500 ms after sentence end for young participants, and for 3000 ms for old participants, after which it disappeared and the trial ended. The timeout for answering the question was the clearing of Display 2. The experiment comprising the administration of the cognitive tests and the eye-tracking experiment lasted on average 45 minutes for the young participants, and about one hour or longer for the older participants.

## Analyses

### Pre-tests

For each participant, we computed scores for the mood and cognitive tests according to instructions specific to the tests (see Table 1). *T*-tests for independent samples were carried out to test for differences between young and older adults on these variables.

### Eye-tracking data

The analyses of the eye tracking data covered the time from the appearance of the two pictures (see Display 2 in Fig. 1) until the end of the sentence. We analysed participants' fixations on the pictures during the time just before and while they processed the sentence. All trials (i.e., correct and incorrect ones) were included in the analyses. It should be noted that the initial part of the sentence (cf. *Ich meine dass* in Fig. 1) is neutral between the negative and positive sentence condition and that disambiguation towards one or the other picture is only possible starting from the initial NP of the embedded sentence (NP1). Therefore one can distinguish two time periods, before, and after referential disambiguation. We refer to these two time periods as the pre-NP1 (onset) region and the post-NP1 (onset) region respectively. The pre-NP1 region includes, in addition to the initial, neutral part of the sentence, the last 1200 ms of the 1500-ms picture preview pre-sentence onset (cf. Display 2 in Fig. 1). The first 300 ms of this preview period were excluded because during this time most participants' fixations were not on the pictures, but on the space which had been occupied by the face in the preceding (prime) display. The total duration of the pre-NP1 region was 3000 ms. The post-NP1 period comprises the duration of the sentence from the onset of NP1, including only fixations starting after NP1 onset, until the end of the sentence (Average duration 4016 ms, *SD* = 456). A sentence effect (i.e., fixations on the pictures as a function of the sentence being heard), as well as face-sentence priming (i.e., increased fixations on the sentence-congruent pictures when the face is congruent with the sentence, relative to when it is incongruent) can thus only be predicted to occur after the sentence becomes referentially disambiguated, and not before. On the other hand, face-picture priming (i.e., looks to the pictures as a function of prime face) can occur both before and after sentence disambiguation.

The fixation measure for most of the analyses was the mean log gaze probability ratio. This is the log of the ratio of the probability of looking at the picture of the negative event over the probability of looking at the picture of the positive event (*ln*(*p*(neg picture)/*p*(pos picture)). This measure expresses the strength of the visual bias towards the negative picture relative to the positive one. It is particularly suited for eye-tracking data analyses with parametric tests (such as ANOVAs) because it does not violate the independence, and the homogeneity of variance assumptions [68]. The log ratio is symmetrical around zero: A positive log ratio indicates more looks to the negative than the positive picture; a negative log ratio indicates more looks to the positive than the negative picture while a value of zero means the two pictures get an equal number of looks.

For exploratory purposes, we first plotted graphs of the fixation pattern in the pre-NP1 and post-NP1 region for each age group. To determine the global trends pre- and post-NP1, inferential analyses were first carried out on the fixation data of the pre- and post-NP1 region respectively. Because our main interest lies in what happens in the incremental processing of the disambiguating post-NP1 region, we subsequently performed inferential analyses on the separate word regions of the post-NP1 region, namely, NP1, NP2, Adverb and Verb regions (see Table 4 for details).

**Table 4:** Onsets, offsets and average duration of the word regions from NP1 onset (*SD* in parentheses).

Word Region	Start	End	Avg duration in ms
NP1 “*die Mechaniker*”	NP1 onset	NP2 onset	1300 (226)
NP2 “*bei der Explosion*”	NP2 onset	Adverb onset	1100 (267)
Adverb “*hilflos*”	Adverb onset	Verb onset	900 (137)
Verb “*zusehen*”	Verb onset	Verb offset	716 (108)

An additional analysis on the eye tracking data used as a measure the duration of the first fixation (in ms) after the onset of NP1. In eye-tracking research on language processing, this measure is usually considered to be an index of early processing, revealing processes occurring in the very early stages of processing a word [78]. In the case of our experiment, the assumption is that the duration of the first fixation on the visual display after the onset of the first disambiguating word (i.e., NP1) would reflect listeners' initial attempt to relate the current word to the visual elements in the display.

### Reaction time and accuracy

The reaction times (RTs) to answer the question (“Does the face match the sentence?”) were measured from the onset of NP1 (i.e., the start of the disambiguating region) until the time of the button press. Statistical analyses were performed on the data after excluding timeouts and incorrect trials. This data contained 1490 cases (802 for young and 688 for old participants) out of an original total of 1792 (896 per age group). Accuracy scores were computed on a total of 1737 observations, which were the sum of incorrect and correct trials (878 for young and 859 for old after excluding timeout trials).

## Results

### Pre-tests

Table 1 summarizes the results of the pre-tests and the demographic details.

As can be ascertained from the data in Table 1, older people performed significantly worse than young adults only in the Picture Completion and the Digit Symbol tasks of the Wechsler Adult Intelligence Test. In all the other tests, including the BMIS mood rating test, the two groups did not differ significantly from each other.

### Eye movement, response time, and accuracy analyses

We first describe the time course of eye movements to the target pictures, then report the inferential analyses on the eye-movement data before (pre-NP1), after (post-NP1) referential disambiguation, and for individual word regions. Then we present the analyses of first fixation durations after referential disambiguation, and end with the response time and accuracy analyses.

#### Time course graphs

**Figure 2:**
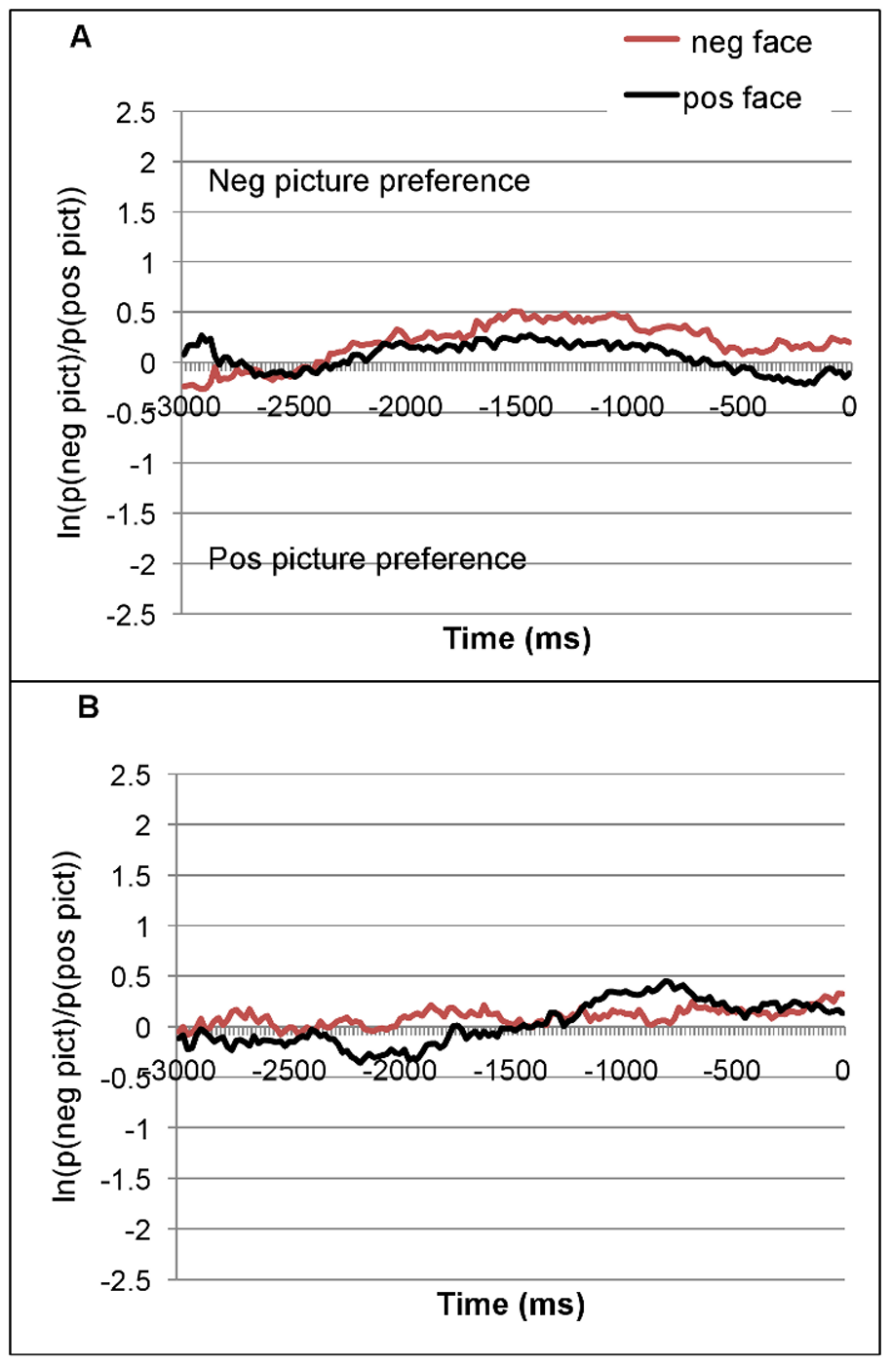
Mean log gaze probability ratios for (A) young and (B) older participants in the pre-NP1 onset region (3000 ms before the onset of NP1). Time 0 =  onset of NP1.

**Figure 3:**
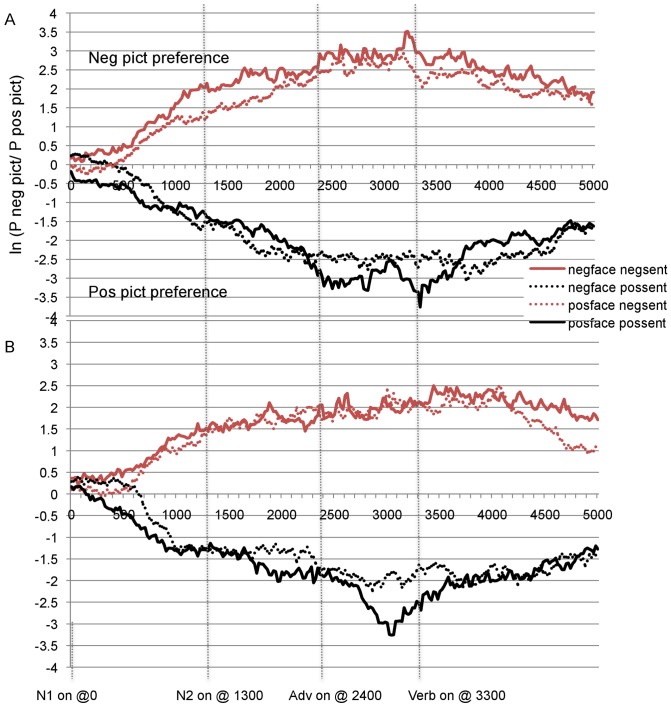
Mean log gaze probability ratios for (A) young and (B) older participants in the post- NP1 onset region.

Fig. 2 (a–b) and Fig. 3 (a–b) plot the time course of fixations to the target pictures for the pre-NP1 and the post-NP1 regions respectively for the two age groups. These graphs are based on mean log gaze probability ratios (henceforth ‘log ratios’) computed on successive 20 ms time slots. In Fig. 2 the log ratios are plotted as a function of prime face, while in Fig. 3 they are plotted as a function of prime face and sentence valence. In Fig. 2 we see a face-picture priming effect (there are more looks to the negative picture after a negative than positive prime, indicated by the red line lying above the black line). For the younger adults (Fig. 2–a) this effect was more extended in the pre-NP1 region than for the older adults (Fig. 2–b). Furthermore, the younger group overall preferred to look at the negative picture (both lines lie above zero for the central 2000-ms portion). For older people, the pattern differed: A face-picture priming effect emerged in the first 1500 ms, then the pattern reversed.

In Fig. 3 (a–b) for the post-NP1 region the sentence effect is apparent in the two sets of lines separating from about 500 ms after NP1 onset: The red lines (representing the two negative sentence conditions) rise steadily above zero, indicating an increasing preference for the negative picture; by contrast, the black lines (representing the positive sentence conditions) go in the opposite direction, indicating an increasing preference for the positive picture. The influence of the prime face on the processing of the sentence (the face-sentence priming effect, i.e., the predicted facilitatory effect occurring from having seen a congruent face) is seen in the relative distance between the solid and the dotted line of each sentence condition: Having seen a face of the same valence as the sentence facilitated the processing of the sentence, as indicated by a greater absolute value for the congruent (solid line) than the incongruent condition (dotted line).

#### Pre- and post-NP1 region

Mean log ratios for the pre-NP1 and post-NP1 region (by averaging over gaze probabilites in successive 20 ms time slots) were computed according to condition by participants and items. Repeated measures ANOVAs were performed on these means, with participants and items as random effects [82]. For the pre-NP1 region the fixed factors were Prime face and Age, while for the post-NP1 region, the fixed factors were Prime face (positive vs. negative), Sentence (positive vs. negative) and Age (young vs. old). Prime face and sentence were within-participants and -items factors in the participant and item analysis, while age was between in the participant and within in the item analysis. Note that because our dependent measure expresses a ratio (of looks to one picture relative to the other), picture (negative vs. positive) is *not* a factor in these ANOVAs. Therefore in these analyses a main effect of Face reflects face-picture priming while a Face x Sentence interaction indicates face-sentence priming. In turn, a Face x Sentence x Age interaction indicates a modulation of face-sentence priming by age.

The ANOVA on the pre-NP1 region confirmed a significant effect of Face (*F1*(1,62)  = 6.44, *p* = .014; *F2* (1,27)  = 8.43, *p* = .007): Participants fixated the negative picture more when the face was negative (*M* = .15, *SE* = .03) than when it was positive (*M* = .04, *SE* = .03). This effect did not interact with age (*F1*(1,62)  = 1.1; *F2*(1,27)  = .84)). The intercept (the grand mean of the log ratio, .094, *SE* = .02) was also significantly different from zero (*F1*(1,62)  = 18.76, *p* = .000; *F2* (1,27)  = 6.48, *p* = .017). Because a log ratio of zero means no preference for either picture, a significant intercept with a positive grand mean indicates that during the pre-NP1 region there were significantly more looks to the negative than the positive picture (irrespective of prime face). However, the visual bias for the negative picture was weaker for older (*M* = .05, *SE* = .03) than younger people (*M* = .13, *SE* = .03). This difference was responsible for a marginal effect of Age in the participants' analysis (*F1*(1, 62)  = 3.44, *p* = .069; (*F2*(1,27)  = 1.9, *p* = .18). In sum, in the pre-NP1 region participants inspected the negative picture more after seeing a negative (vs. positive) prime face; they inspected the negative picture more than the positive picture; and the latter bias was somewhat reduced in older (vs. younger) adults.

The ANOVAs on the post-NP1 region revealed a significant effect of Face (*F1*(1, 62)  = 14.69, *p* = .000; *F2* (1,27)  = 32.38, *p* = .000), with a negative picture preference when the face was negative (*M* = .15, *SE* = .05) and a positive picture preference when the face was positive (*M* = −.11, *SE* = .04). This effect was not modulated by age (both *F*'s <1). As expected, there was a significant sentence effect (*F1*(1,62)  = 934.32, *p* = .000; *F2*(1,27)  = 500.30, *p* = .000): When the sentence referred to the negative picture, participants looked at the negative picture (*M* = 1.65, *SE* = .05), and the opposite was true when the sentence was positive (*M* = −1.61, *SE* = .07).

The 3-way Face x Sentence x Age interaction was fully significant by participants (*F1*(1,62)  = 5.24, *p* = .025; *F2* (1,27)  = 2.45, *p* = .13). For our experimental hypotheses, this interaction is crucial to get at the facilitating effect of the face on the processing of the sentence (face-sentence priming) and the possible modulation of this effect by age. To assess the nature of the differences underlying the Face x Sentence x Age interaction, for each age group, we compared (post-hoc pairwise) the two negative sentence conditions (condition (a) vs. (c) of Table 3), and the two positive sentence conditions (condition (b) vs. (d) of Table 3), applying the Bonferroni adjustment for multiple (4) comparisons (.05/4 = .0125, new corrected *alpha* level). These comparisons can tell us if the valence of the face affects younger and older participants' processing of the sentence differently. For the younger participants' data, the difference between the two negative sentence conditions (neg face-neg sent *M* = 2.00, *SE* = .08 vs. pos face-neg sent *M* =  1.63, *SE* = .09) was significant (*t1*(31)  = 2.68, *p* = .012; *t2*(27)  = 3.1, *p* = .005), but the difference between the two positive sentence conditions (neg face-pos sent *M* = −1.70, *SE* = .11 vs. pos face-pos sent *M* = −1.84, *SE* = .11) was not (*p1*  = .26; *p2*  = .07). The corresponding comparisons for the older group yielded opposite results: The two positive sentence conditions (neg face-pos sent *M* = −1.22, *SE* = .11 vs. pos face-pos sent *M* = −1.66, *SE* = .12) differed significantly (*t1*(31)  = 4.15, *p* = .000; *t2*(27)  = 2,65, *p* = .013), but the negative conditions (neg face-neg sent *M* = 1.54, *SE* = .12 vs. pos face-neg sent *M* = 1.44, *SE* = .11) did not (*p1*  = .49; *p2*  = .17). These results show that for the younger participants a negative (vs. positive) prime face significantly enhanced looks to the negative picture during the processing of a negative sentence, while a positive face did not enhance the processing of a positive sentence. For the older group however, the opposite occurred: A negative face had no effect on the processing of a negative sentence, but a positive (vs. negative) face triggered more looks to the positive picture when the positive sentence was processed.

In the ANOVA on the post-NP1 region there was also a Sentence x Age interaction (*F*1(1,62)  = 9.45, *p* = .003; *F*2 (1,27)  = 135.87, *p* = .000). However, this interaction is not equivalent to a valence x age interaction, but represents an age effect which is not relevant to our research question. The interaction reflects that older adults have less extreme log-ratio values than young adults (i.e., the older adults made fewer fixations to the target pictures than the young adults). Further information about how this interaction arises is given in the Supporting Information S3.

To assess the time course of face-sentence priming for each age group at particular times during the processing of the sentence, we computed mean log ratios relating to the individual word regions, i.e., NP1, NP2, Adverb and Verb (see Table 4 for details of these regions) and performed pairwise comparisons similar to the ones reported above for the whole post-NP1 region (4 comparisons per word region, Bonferroni adjustment: .05/4 = .0125, corrected *alpha* level).

For the NP1 region, the comparisons confirmed previous analyses, i.e., older participants showed a facilitation from the positive prime face in the positive sentence conditions (*t1*(31)  = 3.67, *p* = .001; *t2*(27)  = 2.57, *p* = .016; pos face-pos sent *M* = −.84, *SE* = .14; neg face-pos sent *M* = −.34, *SE* = .09) but not in the negative sentence conditions (*t1*(31)  = 1.68, *p* = .10; *t2*(27)  = 1.81, *p* = .081; neg face-neg sent *M* = .87, *SE* = .12; pos face-neg sent *M* = .60, *SE* = .49), and young participants showed facilitation in the negative sentence condition (*t1*(31)  = 3.58, *p* = .001; *t2*(27)  = 3.60, *p* = .001; neg face-neg sent *M* = 1.17, *SE* = .12, pos face-neg sent *M* = .64, *SE* = .08), but only marginally in the positive sentence condition (*t1*(31)  = 1.93, *p* = .06; *t2*(27)  = 2.46, *p* = .02; neg face-pos sent *M* = −.61, *SE* = .13; pos face-pos sent *M* = −.96, *SE* = .12). The only other significant comparison occurred in the adverb region for the positive sentence conditions with older participants (*t1*(31)  = 2.35, *p* = .025; *t2*(27)  = 2.07, *p* = .048; neg face-pos sent *M* = −2.97, *SE* = .40, pos face-pos sent *M* = −3.90, *SE* = .39; *t*-test comparing the negative sentence conditions: *t1*(31)  = −.059, *p* = .95; *t2*(27)  = −.30, *p* = .76). This confirms that for older adults a positive face, compared to a negative one, facilitated the processing of a positive sentence, but a negative face was of no advantage in processing a negative sentence.

In sum, the ANOVAs and the comparisons on the data of the post-NP1 onset region revealed that participants of both ages looked more at the picture that was of the same valence as the prime face, and of the same valence as the sentence. The fact that results were significant in the NP1 region for *both* age groups suggests that the integration of the visual context with facial and linguistic information occurs early, and that its time course does not substantially differ between the two age groups. In line with our original predictions, the Face x Sentence x Age interaction and the follow-up pairwise comparisons show significant facilitation from a congruent prime face in the young group for the negative sentence, and in the older group for the positive sentence. This last result supports the claim that older people focus more on positive information and younger ones on negative information. Note that the interactions of prime face with sentence cannot be accommodated by appealing merely to a strategy of inspecting the picture that matches the valence of the prime face. The sentence *mismatched* the face valence for half of the experimental items a participant saw, and thus merely inspecting the face-valence matching picture without integrating sentence meaning would lead participants to inspect the incorrect target picture on half of the experimental trials. This design property permits us to be certain that face-sentence interactions reflect effects of the facial prime on *sentence* processing.

#### Duration of 1^st^ fixation after the onset of NP1

We submitted the first fixation duration data to repeated measures ANOVAs, where the fixed factors were fixated picture (positive vs. negative), prime face (positive vs. negative), sentence (positive vs. negative) and age (young vs. old). Picture, face and sentence were within-participants and -items factors in the participant and item analysis, while age was between in the participant and within in the item analysis.

**Figure 4:**
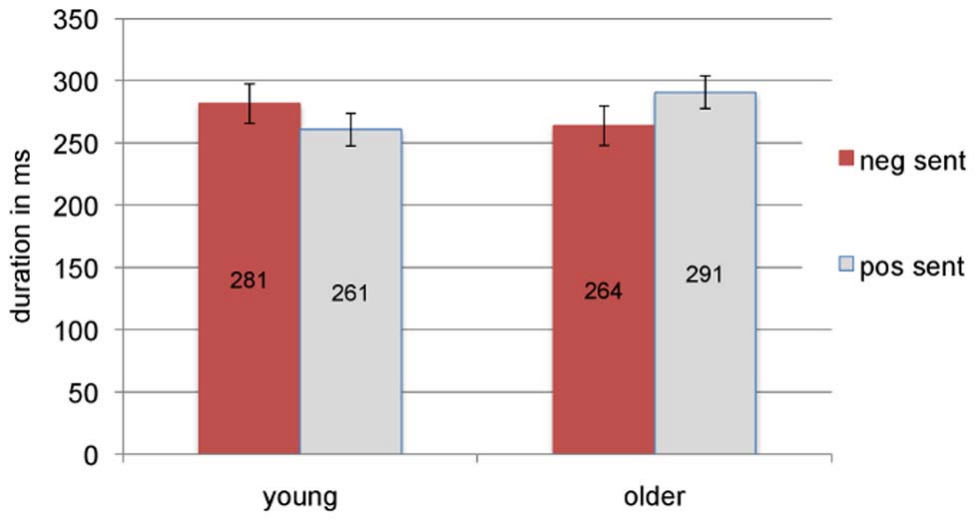
Sentence x Age interaction in first fixation duration after NP1 onset, with standard errors.

None of the main effects were significant; however, the Picture x Face interaction was significant by participants (*F1*(1,62)  = 9.02, *p* = .004; *F2* (1,27)  = 1.31, *p* = .26). It resulted from longer fixation durations when face and picture were emotionally congruent (vs. incongruent). This interaction corroborates the face-picture priming effects in the pre-NP1 and in post-NP1 regions at the very local level, while processing the early part of NP1. Of particular interest is the Sentence x Age interaction (*F1*(1,62)  = 4.62, *p* = .036; *F2* (1,27)  = 6.24, *p* = .019). As one can see from Figure 4, for young participants mean first fixation durations are longer when the sentence is negative (281ms, *SE* = 19.2 vs. 261ms, *SE* = 12.2), while for the older participants they are longer when the sentence is positive (291ms, *SE* = 14.0 vs. 264ms, *SE* = 11.8). Pairwise comparisons on the means for the two groups revealed a significant difference for the older (*t1*(31)  = −2.03), *p* = .05; *t2*(27)  = −2.18, *p* = .04), but not for the younger adults (*t1*(31)  = 1.17, *p* = .25; *t2*(27)  = 1.15, *p* = .26). A plausible interpretation of this interaction is that, for older people, positive (vs. negative) sentences act as a trigger to inspect the visual display in more depth (hence the longer fixation durations), while younger participants' first fixation durations are not selectively affected by sentence valence. This finding is in line with the general prediction of a positivity effect for older adults. Note that the 3-way Sentence x Age x Picture interaction was not significant, therefore the pattern of the observed Sentence x Age interaction is independent of the picture that was fixated.

#### Reaction times

**Figure 5:**
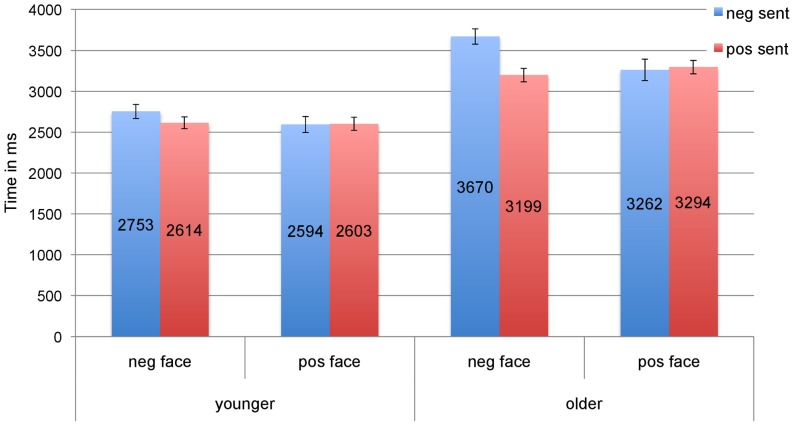
RTs: Reaction times in ms with standard errors, measured from NP1 onset, by condition and age group.

The RT data was submitted to 3-way repeated measures (Face x Sentence x Age) ANOVAs similar to those on the eye tracking data (see Figure 5). A main effect of age (*F1*(1,62)  = 46.25, *p* = .000; *F2*(1,27)  = 385.40, *p* = .000) confirmed that older participants had overall much longer RTs (3356 ms, *SE* =  74.4 vs. 2640 ms, *SE* = 74.4). This was expected, and is in line with prior research (e.g., [64]–[65]). Both the main effects of face and sentence were significant (Face: *F1*(1,62)  = 6.57, *p* = .013; *F2*(1,27)  = 11.81, *p* = .002; Sentence: *F1*(1,62)  = 8.70, *p* = .004; *F2*(1,27)  = 4.57, *p* = .042), with negative face primes and negative sentences triggering longer RTs than their positive counterparts. Importantly, these effects were qualified by two interactions, one involving Age. First, there was a significant trend in the analysis by items towards a Sentence x Age interaction, (*F2*(1,27)  = 5.71, *p* = .024; *F1*(1,62)  = 2.56, *p* = .11). By-age pairwise comparisons showed that older people responded significantly faster when the sentence was positive than when it was negative (3247ms, *SE* = 69.5 vs. 3466 ms, *SE* = 97.0; *t1*(31)  = 2.94, *p* = .006; *t2*(27)  = 2.56, *p* = .016); however, for younger adults this difference (2608ms, *SE* = 67.6 vs. 2673ms, *SE* = 89.3) was not significant (*t1*(31)  = 1.06, *p* = .29; *t2*(27)  = .83, *p* = .41). The Face x Sentence interaction was reliable (*F1*(1,62)  = 17.85, *p* = .000; *F2*(1,27)  = 14.11, *p* = .001), and was significantly modulated by Age, i.e., Face x Sentence x Age interaction: (*F1*(1,62)  = 5.29, *p* = .025; *F2*(1,27)  = 7.06, *p* = .013). By-age pairwise comparisons revealed that older adults responded significantly more slowly in the congruent than incongruent negative face condition (*t1*(31)  = 5.48, *p* = .00; *t2*(27)  = 5.13, *p* = .00), and the same was true for younger adults in the comparison by participants (*t1*(31)  = 2.07, *p* = .04; *t2*(27)  = 1.53, *p* = .13; *n.s.* in positive face conditions in either group). Figure 5 shows that these interactions are carried by the negative face-negative sentence condition, which triggered the slowest RTs in both age groups. The Face x Age interaction did not reach significance (both *F*'s <1).

The Sentence x Age interaction could be interpreted as a positivity effect (older adults are faster than younger adults with positive than negative sentences), however the same interpretation appears problematic for the Face x Sentence x Age interaction because young adults' behavior vis-à-vis the congruent negative face condition is similar to older adults'; in fact, the overall RT pattern is similar for young and old (Figure 5). We return to this in the discussion (next section).

#### Accuracy

**Figure 6:**
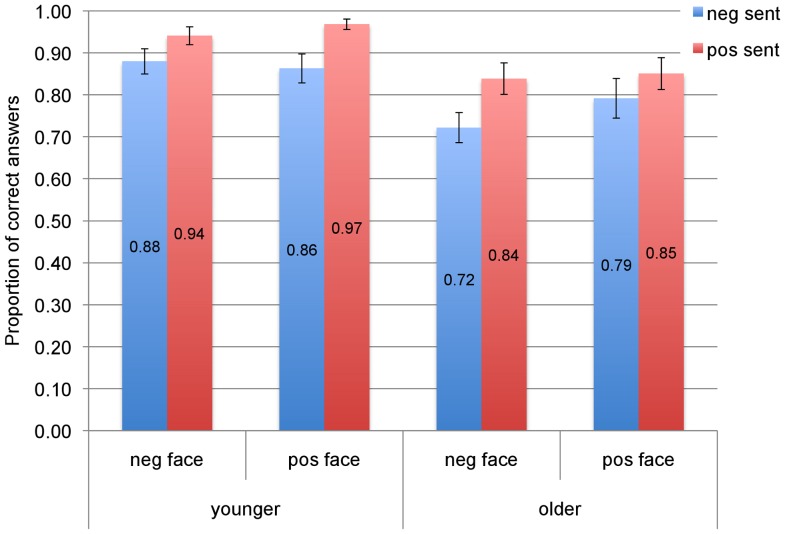
Proportions of correct answers to verification question, with standard errors.

The proportions of correct answers as a function of face and sentence valence are illustrated in Figure 6 for the two age groups. We fitted a logistic linear mixed effect (LME) model to the binary (i.e., correct vs. incorrect) response data [83]. In this model the predicted outcome was the response and the predictors were face and sentence valence, each with two levels (negative vs. positive), and age (young vs. old). Participants and items, with their intercepts and slopes and the intercept x slope interactions, were included in the random effects part of the model. We transformed the fixed effect coding of the predictors into a numerical value and centered it so as to have a mean of 0 and a range of 1 [84]. This effect coding has the advantage of reducing collinearity and allows the coefficients of the regression to be interpreted as the main effects in a standard ANOVA [85]. The results of the model are given in Table 5.

**Table 5:** Linear mixed effect model results for accuracy scores.

	*Coefficient*	*SE*	*z-Value*	*p*
***Intercept***	2.46	.16	15.1	.000
***Face valence***	.34	.10	3.36	.000
***Sentence valence***	.53	.15	3.54	.000
***Age***	.68	.10	6.76	.000
***Face x Sent***	.19	.13	1.05	.29
***Face x Age***	.10	.11	.88	.38
***Sent x Age***	.19	.11	1.60	.11
***Face x Sent x Age***	.17	.10	1.85	.06

As one can observe from Table 5, all three main effects were significant. Accuracy scores were higher for the positive face and positive sentences than their negative counterparts. The age effect indicates that older people were overall less accurate in their answers. Similar to longer RTs, lower accuracy scores for older compared to younger populations are expected [72]–[73]. Furthermore the Face x Sentence x Age interaction was marginally significant (*p* = .06, cf. the blue bars in Fig. 6): In the negative sentence conditions, the negative (vs. positive) face did not appear to affect accuracy in the young group (*M* = .88, *SE* = 0.03 vs. *M* = .86, *SE* = 0.03); however, in the older group, a positive (vs. negative) face increased accuracy (*M* = .72, *SE* = .04 vs. *M* = .79, *SE* = .05). This last result is compatible with the hypothesis that older people focus more on positive than negative information. However, as for the RTs, the accuracy results appear broadly-speaking similar for young and older adults (see Fig. 6) in that both groups had lower accuracy for negative sentences and negative faces.

## Discussion

### Eye tracking results

The eye tracking results provide evidence that emotion priming occurs *during* sentence processing: Participants' looks to the relevant picture in the visual display were enhanced when the sentence was emotionally congruent (vs. incongruent) with a preceding prime face. This enhancement was observed in the earliest region where sentence disambiguation was considered possible (NP1), suggesting that the integration of emotionally congruent information from the facial, visual and linguistic modalities happens incrementally. To our knowledge, these results are the first to demonstrate emotion priming during incremental sentence interpretation.

Participants further tended to look at the picture that was emotionally congruent with the face (face effect). Finding this effect in the pre-NP1 region shows that emotional information from the face prompted *anticipatory* looks to the picture (i.e., in anticipation of the sentence) that was emotionally face-congruent. This effect continued into the processing of the disambiguated part of the sentence (Picture x Face interaction in first fixation durations and face effect in the post-NP1 region).

With regard to the age manipulation, the eye-tracking results revealed differences in the processing of emotional information between younger and older adults (post NP1). For older adults a congruent emotional face facilitated the processing of positive sentences only; by contrast, for younger adults facilitation emerged when processing negative sentences (or both negative and positive sentences). The analysis of first fixation durations after NP1 onset provides additional evidence for young and older adults' different early visual attention bias, with older adults making longer fixations for positive and young adults for negative sentences. The fact that this age-dependent effect surfaced in an early processing measure is important for its characterization (of which more in the General Discussion). Prior to sentence disambiguation, the eye tracking results also showed that the bias to look at the negative picture was reduced for older adults compared to younger ones. Taken together, these findings are compatible with the presence of a positivity effect for older adults, both in the direction of an increased eye-movement response to positive stimuli and that of a reduced attention response to negative ones.

### Reaction times and Accuracy results

One important conclusion that can be drawn from the RT and accuracy results is that matching a negative sentence with a (negative) face was more difficult than decisions involving a positive sentence. This difficulty occurred for both age groups, although to a greater extent for the older participants. One possible explanation why both age groups found the combination of a negative face with a negative sentence particularly difficult could lie in the nature of sad faces –sad faces seem more difficult to identify than happy ones [26],[86]. Note that verifying the mismatch of a sad face with a positive sentence should not cause the same difficulty, as in this case a sad facial expression (even a not so optimal exemplar) and a positive sentence would be perceived more readily as being “different”. However, this explanation (that sad faces are more difficult to recognize than happy faces) appears to be in contrast with the eye tracking results. Arguably, the significant face effects in the eye-movement record demonstrate that participants did not delay launching looks to the negative picture after seeing a sad face, and this suggests that a sad face was easily and readily identified as negative.

Another disparity between the RT and accuracy results compared with the eye-movement results is that, the latter revealed the predicted facilitation in face-sentence congruent compared to incongruent trials (and its modulation by age), but the RT and accuracy results did not. A plausible conclusion that can be drawn from this is that the eye-tracking results reflect initial, incremental processing of the sentence in relation to the face and the pictures, while the verification results reflect post-encoding processes ([87], but see [70]). We return to this in the general discussion.

## General Discussion

This study had two concurrent aims, i.e., to investigate emotion priming in sentence comprehension and its time course, and to assess whether and to what extent age affects this process. Taking as a starting point previous findings that priming from emotional faces occurs for words, we hypothesized that it might also occur during incremental visually-situated sentence interpretation. We addressed this empirical question using the visual world eye-tracking paradigm, a methodology that provides a fine-grained millisecond-to-millisecond record of how a listener understands a sentence in relation to the concurrent visual context.

In our experimental design, for emotion priming of sentence processing to occur, two sources of visual information (the speaker's emotional face and the emotional pictures) had to be integrated with the linguistic information from the sentence. One hypothesis was that this integration may be cognitively demanding and that priming may not occur incrementally but be delayed, perhaps surfacing only in post-processing stages. The alternative hypothesis was that emotion priming occurs on the fly and influences sentence comprehension in a visual context as the utterance unfolds. The latter hypothesis was partly motivated by previous evidence that humans attend to emotional (vs. neutral) stimuli faster, and process them more deeply. In line with this, the effects of an emotional face on sentence processing should surface, if not at the first available opportunity, then at least before the end of the sentence. Our results support the latter hypothesis. Face-sentence priming (facilitation in the face-sentence congruent conditions) emerged at the earliest word region (NP1) where such facilitation was considered possible. To our knowledge, these findings are the first to demonstrate emotion priming during sentence comprehension in a visually-situated task (i.e., when language is about objects and actions in the visual context).

With regard to the age manipulation, both the eye tracking and some of the verification task results revealed significant differences between younger and older adults. Older adults exhibited a reduced focus on negative stimuli compared to younger adults and an attention bias towards positive information. On the other hand, younger adults displayed an attention bias towards negative information. All the age differences that we observed are consistent with the broader definition of the positivity effect predicted by socioemotional selectivity theory [50],[52]. To our knowledge, these results are the first to show positivity effects in real time during incremental sentence processing. Interestingly, it is in the eye-movement findings that positivity effects surfaced in a clear and straightforward way. Altogether, in the eye movement data there were three instantiations of a positivity effect, two of which were corroborated by fully significant statistical results and the third by a marginal result. In the first two instantiations (the Face x Sentence x Age interaction, and the follow-up comparisons in the post NP1 region, and the Sentence x Age interaction in first fixation durations) older and younger adults showed asymmetric behavior with regard to processing emotional information: Older adults were influenced more by positive information (from the face or sentence) and younger adults by negative information. In the third instantiation of the positivity effect (the age effect in the pre-NP1 region), older participants showed a reduced focus on the negative IAPS pictures compared to younger participants. Thus, these positivity effects are not limited to processing emotional information in a particular modality (e.g., only faces, pictures or language) but cover different modalities through which emotional information can be expressed.

### The speaker as context

Overall, our results corroborate and extend previous evidence on the interaction of the visual context with sentence processing, in particular, with regard to the role that speaker-based cues play in such processing. Previous research has demonstrated that speaker-based information such as gaze and gestures are rapidly integrated into sentence processing. Importantly, our results provide evidence that a speaker's emotional facial expression also has a rapid influence on sentence interpretation. Furthermore, the time course of this influence appears to be similar to the time course of the effect of speaker gaze, as demonstrated, for example, in the already-mentioned study by Kreysa and Knoeferle [18]. In that study, too, the effect from the speaker's gaze occurred very early, in that listeners were able to make anticipatory eye movements to a postverbal referent *earlier* when the speaker's gaze was available than when it was not (see also [14]). That speaker gaze and facial expression should exert an early influence on sentence processing is not surprising given the special role that the human face plays in communicating the speaker's intentions and mental state. In this regard, an important question for future research is how a listener's visual attention is affected when both cues, i.e., the speaker's facial expression and his/her gaze, are available at the same time.

We have so far assumed that the finding of a face priming effect on sentence processing (as reflected in eye movements by a significant Face x Sentence interaction) shows that a *speaker*'s emotional face facilitates the processing of an emotionally-congruent sentence. Arguably, the validity of this assumption depends on how successful we were in simulating a speaker-hearer scenario and on the extent to which our participants associated the face with that of the imaginary speaker of the sentence. Even if participants were told before the experiment to imagine that the face on the screen was the face of the speaker of the sentence they were about to hear, it could be argued that the observed facilitatory effect on sentence processing stems from a simple emotion priming mechanism similar to those found in more traditional emotion priming experiments (see Introduction). We think this interpretation is possible but unlikely. What makes it unlikely is the fact that a significant main effect of Face was observed not only in the pre-NP1 region, but also in the post-NP1 region. In the pre-NP1 region the emotional valence of the face could be used, and was indeed used by participants (hence the main effect of Face), as a cue predicting which picture the future sentence was about. However, in the post-NP1 region when the sentence was being processed the face could no longer act as a valid predictor cue, since the sentence matched the face in valence only half of the time. Despite this, and despite the occurrence of a Face x Sentence interaction, the effect of Face persisted in the post-NP1 region. We suggest that this persistence shows that participants were still expecting that the sentence would be emotionally congruent with the face; in particular, they were expecting what is normally expected of a real human speaker, and not just any face, i.e., that her facial expression will be consistent with the emotional content of her sentences.

Related to the above, a more immediate speaker-hearer scenario could have been achieved by presenting the face at the same time as the sentence and the IAPS pictures. Our decision to present the face separately was motivated by maximizing the likelihood of fixations on the IAPS pictures. Another factor detracting from ecological validity is the use of neutral intonation for our emotional auditory sentences: In real life, emotional speech is usually uttered with typical emotional prosody, a contextual cue that has been shown, for example, to aid in the recognition of facial emotions [59]. We used neutral prosody to rule out confounding factors for the effects of our priming variable (the facial prime).

### Reconciling the eye-movement and post-sentence results

Although interactions involving valence and age emerged in the verification-task results, as we noted these do not directly reflect the eye-gaze pattern. In particular, the difficulty encountered by both younger and older adults in verifying negative sentences precludes concluding that a clear positivity effect also occurred in this measure. The divergence in the eye-tracking and verification data, and the fact that a clear positivity effect was only observed in the eye-tracking data and in early processing (i.e., in the very first critical region, the NP1 region), has interesting implications for the mechanisms implicated in the positivity effect, and the level(s) of processing at which they operate. Indeed, this has been one of the issues under discussion in the framework of socioemotional selectivity theory. A central tenet of this theory is that emotion regulation improves with age and that the positivity effect occurs because older people are capable (consciously or unconsciously) to selectively regulate their emotions in order to enhance positivity and well-being; thus, according to this view, the positivity effect should be strongest in tasks and situations that require controlled processing with associated exertion of cognitive effort, and less so in tasks that measure automatic or initial processing (see [44] p. 137).

Indeed, evidence from several studies suggests that positivity effects depend on deliberate mood regulation strategies [88]–[90]. When cognitive control resources are constrained (e.g., as in divided attention tasks) positivity effects do not emerge [91]–[92]. Furthermore, the strength of these effects has been shown to be positively correlated to measures of cognitive control/executive functioning in older adults [89], [92]. However, recent evidence suggests that controlled processing and cognitive effort are not *necessary* to trigger positivity effects in older adults. For example, older adults exhibit positivity effects in divided attention tasks [93]–[94], and when suffering brain lesions in pre-frontal cortex, an area of the brain normally associated with cognitive control [95]. In a task in which participants viewed facial expressions of different emotions, and using pupil dilation as a measure of cognitive effort, Allard et al. [96] found that older adults' positive gaze preferences were not associated with increased cognitive effort. This evidence is compatible with the view that positivity effects in older adults may, under certain circumstances, be due to an automatic mechanism of emotion processing rather than a mechanism requiring strategic regulation of emotion.

We suggest that the positivity effects found in the eye tracking measures of our experiment are more likely to be a result of an early, non-strategic emotion processing mechanism. One reason for this is the time course associated with these effects in our experiment: As we already noted, these effects were first observed in the early processing stages of the sentence (NP1 region) and in early measures of processing (first fixation duration after NP1 onset). In eye-tracking research in psycholinguistics, effects observed in the early stages of processing are usually ascribed to non-strategic processes. Furthermore, it is unlikely that the positivity effects observed might have been caused by mood differences between the two age groups. In our experiment mood was not manipulated and, although in the pre-experiment BMIS questionnaire older participants showed a more positive mood overall than younger participants (10.5 vs. 8.34, see Table 1), the mean mood rating was positive for both age groups and the difference was not statistically significant.

Further research is needed to clarify the timing and the nature of positivity effects under different experimental conditions using time-sensitive methodologies. Overall, however, these are the first findings to provide clear evidence for rapid priming of emotions from faces to visual attention during sentence comprehension and for subtle modulation of these early effects by comprehenders' age.

## Supporting Information

Supporting Information S1 **Image files (negative, neutral, positive) of the two faces associated with the experimental sentences, with mean rating and SD scored in the norming study (** ***N*** **= 15); rating scale: 1 =  very negative, 9 =  very positive.**
(PDF)

Supporting Information S2 **IAPS pictures used in the experiment in each item pair and the German sentence associated with each picture.**
(PDF)

Supporting Information S3 **Sentence x Age interaction in the Eye-tracking analyses of the post-NP1 region: How this interaction arises and what it reflects.**
(PDF)
