# Supplementary material for: Effects of Speaker Emotional Facial Expression and Listener Age on Incremental Sentence Processing
Source: PLoS One. 2013 Sep 6;8(9):e72559. doi: 10.1371/journal.pone.0072559 (PMC3765193; doi:10.1371/journal.pone.0072559)
Supplement: Supporting Information S1 — Image files (negative, neutral, positive) of the two faces associated with the experimental sentences, with mean rating and SD scored in the norming study ( N = 15); rating scale: 1 = very negative, 9 = very positive. (PDF) [file pone.0072559.s001.pdf]

Image files (negative, neutral, positive) of the two faces associated with the experimental sentences, with mean rating and *SD* scored in norming study *(N= 15)*; rating scale: 1= very negative, 9 = very positive.

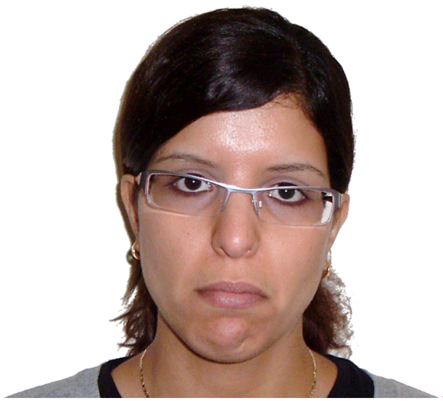

2.94 (1.00)

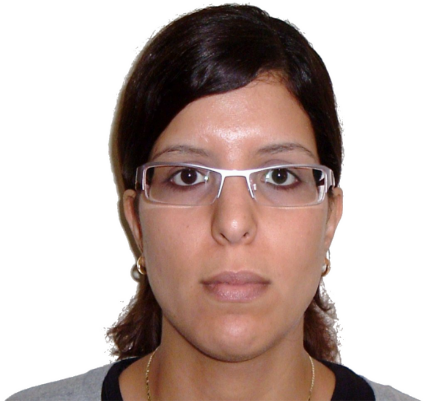

4.78 (1.00)

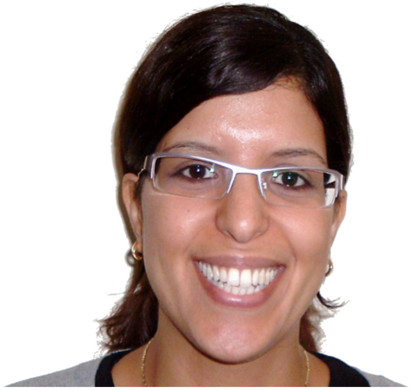

8.39 (.98)

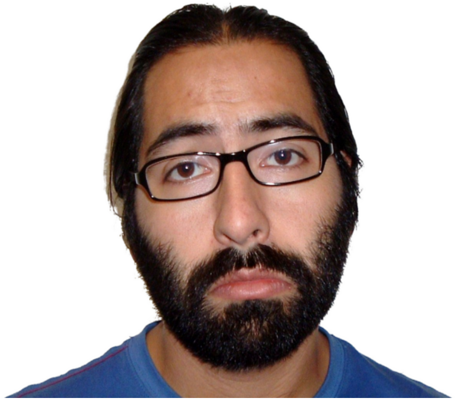

2.89 (1.18)

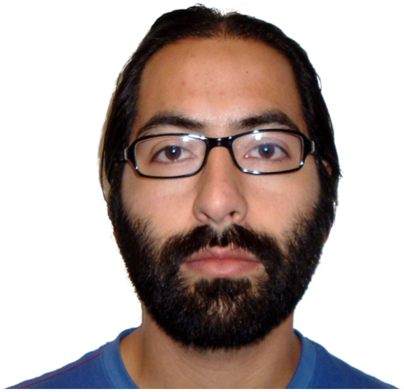

4.39 (.92)

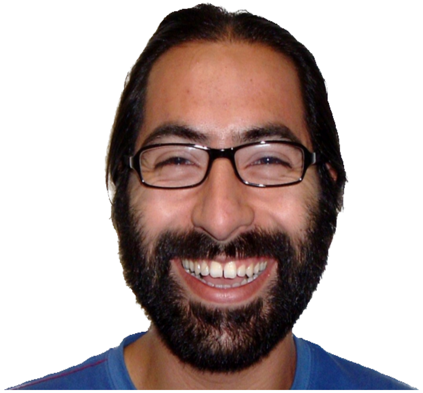

8.28 (.98)
