# Supplementary material for: Effects of Speaker Emotional Facial Expression and Listener Age on Incremental Sentence Processing
Source: PLoS One. 2013 Sep 6;8(9):e72559. doi: 10.1371/journal.pone.0072559 (PMC3765193; doi:10.1371/journal.pone.0072559)
Supplement: Supporting Information S2 — IAPS pictures used in the experiment in each item pair and the German sentence associated with each picture. (PDF) [file pone.0072559.s002.pdf]

IAPS pictures used in the experiment in each item pair and the German sentence associated with each picture.

| Item number | IAPS # of positive picture + sentence | IAPS # of negative picture + sentence |
|---|---|---|
| 1 | 7502<br>Es ist wahrscheinlich, dass die Menge die Luftballons friedlich bestaunt. | 9423<br>Es ist wahrscheinlich, dass die Gruppe die Geiselhaft nervös durchsitzt. |
| 2 | 7325<br>Es ist offensichtlich, dass die Kleine die Melone heiter verspeist. | 2399<br>Es ist offensichtlich, dass die Blonde die Migräne gereizt verflucht. |
| 3 | 4621<br>Ich vermute, dass die Verlobte die Beeren spontan vernascht. | 8232<br>Ich vermute, dass der Boxkämpfer die Schlappe passiv erträgt. |
| 4 | 4626<br>Ich meine, dass die Frischvermählten die Liebe spontan besiegeln. | 6211<br>Ich meine, dass die Guerrilleros die Rache aggressiv ausleben. |
| 5 | 4622<br>Es ist sicher, dass der Spaziergänger den Strandurlaub entspannt verbringt. | 6571<br>Es ist sicher, dass der Autofahrer den überfall ängstlich übersteht. |
| 6 | 8200<br>Ich halte es für plausibel, dass der Sportler das Wasser ausgeglichen durchfährt. | 9341<br>Ich halte es für plausibel, dass der Junge das Flussbett angeekelt durchquert. |
| 7 | 2154<br>Es ist offensichtlich, dass der Sprößling nach dem Kräftemessen fröhlich lacht. | 2456<br>Es ist offensichtlich, dass die Alte nach der Katastrophe furchtsam seufzt. |
| 8 | 4603<br>Ich bin sicher, dass die Gattin das Sträußchen erfreut annimmt. | 3280<br>Ich bin sicher, dass der Patient den Bohrer reizbar duldet. |
| 9 | 4505<br>Ich halte es für plausibel, dass der Mann seine Model-Karriere kreativ verfolgt. | 2752<br>Ich halte es für plausibel, dass die Frau ihre Alkoholsucht feige hinnimmt. |
| 10 | 2091<br>Ich nehme an, dass die Schulkinder die Hauskätzchen zärtlich knuddeln. | 9905<br>Ich nehme an, dass die Brandschützer die Verletzten besorgt bergen. |
| 11 | 5629<br>Ich schätze, dass der Mann den Alpengipfel ehrgeizig bewundert. | 9210<br>Ich schätze, dass die Frau den Dauerregen depressiv betrachtet. |
| 12 | 2216<br>Ich meine, dass die Vorstadtkinder bei dem Badespaß verspielt rumplanschen. | 8485<br>Ich meine, dass die Mechaniker bei der Explosion hilflos zusehen. |
| 13 | 5833<br>Ich schätze, dass die Bevölkerung den Sonnenschein gemütlich auskostet. | 9002<br>Ich schätze, dass die Menschenmenge den Amoklauf bekümmert betrauert. |
| 14 | 4599<br>Ich denke, dass der Jungunternehmer das Rendevous lebhaft zelebriert. | 2039<br>Ich denke, dass die Alleinstehende die Einsamkeit traurig bedauert. |

| | | |
|---|---|---|
| 15 | 4597<br>Ich denke, dass das Mädel die Zärtlichkeit sinnlich findet. | 2590<br>Ich denke, dass die Oma die Arthritis grausam findet. |
| 16 | 5470<br>Ich bin der Meinung, dass der Weltraumfahrer die Pionierfahrt mutig durchlebt. | 9331<br>Ich bin der Meinung, dass der Hilfsarbeiter die Bedürftigkeit beschämt aushält. |
| 17 | 4572<br>Ich halte es für möglich, dass der Feuerwehrmann die Ruhepause angenehm vertrödelt. | 6010<br>Ich halte es für möglich, dass der Verdächtige die Gefangenschaft gelangweilt erduldet. |
| 18 | 4532<br>Ich glaube, dass der Verlobte die Hausparty umsichtig vorbereitet. | 6314<br>Ich glaube, dass der Halbstarke die Schlägerei feindselig herausfordert. |
| 19 | 2370<br>Ich bin überzeugt, dass die Herren das Jubiläum gesellig begehen. | 2455<br>Ich bin überzeugt, dass die Mädchen das Schulmassaker aufgewühlt beweinen. |
| 20 | 4510<br>Ich bin der Meinung, dass der Erwachsene bei dem Pflegebad verträumt enspannt. | 2810<br>Ich bin der Meinung, dass der Rothaarige bei dem Wutanfall zornig zetert. |
| 21 | 5831<br>Ich bin der Ansicht, dass der Vater die Seevögel geduldig anlockt. | 2688<br>Ich bin der Ansicht, dass der Jäger die Eisbären berechnend erschießt. |
| 22 | 2501<br>Es ist sicher, dass das Rentnerpaar die Seereise angeregt verlebt. | 2799<br>Es ist sicher, dass die Familie den Verlust frustriert beklagt. |
| 23 | 2530<br>Ich halte es für möglich, dass die Radfahrer den Sommerausflug amüsiert zubringen. | 9046<br>Ich halte es für möglich, dass die Jugendlichen das Armutsschicksal verärgert befürchten. |
| 24 | 8330<br>Ich vermute, dass der Sportstar die Trophäe höflich empfängt. | 2795<br>Ich vermute, dass der Junge den Todesfall schweigsam verdammt. |
| 25 | 2550<br>Ich nehme an, dass die Rentner bei der Silberhochzeit impulsiv strahlen. | 2278<br>Ich nehme an, dass die Mädchen in der Kältewelle unruhig zittern. |
| 26 | 5829<br>Ich bin der Ansicht, dass die Leute das Strandbaden lustig empfinden. | 2695<br>Ich bin der Ansicht, dass die Passagiere den Flüchtlingstrek müde durchleiden. |
| 27 | 2155<br>Ich bin überzeugt, dass der Gatte die Schwangerschaft einfühlsam wahrnimmt. | 2312<br>Ich bin überzeugt, dass das Kleinkind die Verarmung deprimiert wahrnimmt. |
| 28 | 2398<br>Ich glaube, dass die Familie im Sommerurlaub besonnen ausspannt. | 9403<br>Ich glaube, dass die Einsatztruppe im Kampfmanöver autoritär angreift. |
