# Supplementary material for: Effects of Speaker Emotional Facial Expression and Listener Age on Incremental Sentence Processing
Source: PLoS One. 2013 Sep 6;8(9):e72559. doi: 10.1371/journal.pone.0072559 (PMC3765193; doi:10.1371/journal.pone.0072559)
Supplement: Supporting Information S3 — Sentence x Age interaction in the Eye-tracking analyses of the post-NP1 region: How this interaction arises and what it reflects. (PDF) [file pone.0072559.s003.pdf]

In principle, in an ANOVA a Sentence x Age interaction should indicate that the impact of age depends on the level of the factor Sentence (positive vs. negative sentence), i.e. that the difference between the negative and positive sentence condition is significantly different for young and old. However, in this particular instance, due to the log ratio measure that we are using, this interaction is not equivalent to a Valence x Age interaction. Because the (log ratio) value for the negative condition is a positive number and that of the positive condition is a negative number, the difference as computed by the ANOVA is actually a sum.

If one looks at Fig.3, one can see that this sum corresponds to the distance between the mean of the negative sentence conditions (the two red lines) and the mean of the positive sentence conditions (the two black lines). The distance is smaller for older (Fig. 3b) than for younger adults (Fig. 3a), and this is responsible for the significant Sentence x Age interaction in the ANOVA. The smaller distance for older adults results from their reduced absolute log-ratio values compared with younger participants (independent of the experimental manipulations). The reduced log-ratio values reflect older adults' overall fewer fixations on the critical pictures (they blink more often and have more fixations on the background). Thus, the Sentence by Age interaction in this particular case denotes an age effect which is not relevant to our research aim. This effect is of a similar nature to that observed in ERP studies, where it is found that positive or negative deflections are generally less extreme for older than for younger participants.
